# In vitro regeneration and its histological characteristics of *Dioscorea nipponica* Makino

**DOI:** 10.1038/s41598-022-22986-4

**Published:** 2022-11-01

**Authors:** Shangni Dang, Runmei Gao, Yuqing Zhang, Yumei Feng

**Affiliations:** College of Forestry, Shanxi Agricultural University, Taigu, Shanxi China

**Keywords:** Cell biology, Drug discovery, Physiology, Plant sciences

## Abstract

*Dioscorea nipponica* Makino is an optimal candidate to develop the diosgenin industry in North China. Due to its increasing demand in the medicine industry, it is urgent to apply new biotechnological tools to foster breeds with desirable traits and enhanced secondary metabolite production. The production of useful metabolites by the in vitro cultured rhizomes can be explored successfully for utilization by various food and drug industries. In this study, we reported callus formation and plantlet regeneration of the medicinal plant *D. nipponica*. Explants of leaves, stem segments and rhizomes of aseptic seedlings were cultured on Murashige and Skoog (MS) medium containing various combinations of auxin and cytokinin to find the optimal PGRs of each type of explant for callus induction and shoot regeneration of *D. nipponica*. The paraffin section technique was also used to observe of the morphogenesis of callus and adventitious bud. Explants of seeds and rhizomes formed calli at high frequency in all lines we examined. However, the explant of leaves rarely formed callus. Three kinds of callus were detected during the induction phase. Here, we describe three types of callus (Callus I–III) with different structure characteristics. Greenish in color and a nodule-like protrusion surface (Callus type III) were arranged more closely of cells with less interstitial substance, cell differentiation ability stronger than other callus types. The optimum combination was the maximum shoot differentiation frequency of 90% in callus derived from seeds cultured on MS medium with 2.0 mg L^−1^6-BA + 0.2 mg L^−1^NAA. The shoot differentiation frequency (88.57%) of rhizome-induced callus was obtained by the combination of MS medium supplemented with 3.0 mg L^−1^6-BA + 2.0 mg L^−1^NAA. 1/2 MS medium plus 0.5 mg L^−1^NAA resulted in a higher root regeneration frequency of 86.70%. In vitro propagated plantlets with healthy roots were domesticated and transplanted into small plastic pots containing sterile soil rite under greenhouse conditions with 80% survivability. Bud differentiation is mostly of exogenous origin, mostly occurring on the near callus surface. Therefore, it may be surmised that in vitro morphogenesis of *D. nipponica* is mainly caused by indirect organogenesis (adventitious bud).

## Introduction

The genus *Dioscorea* comprises more than 600 species known for their traditional medicinal properties^1^. *D. nipponica* Makino is a perennial twining herb species. Its rhizome contains the secondary metabolite diosgenin, which is generally used as an important raw material for the production and synthesis of most steroid hormones and contraceptive drugs^2^. Diosgenin has a certain effect on treating coronary heart disease, anti-atherosclerosis, lowering blood lipid, immune regulation, antiasthmatic, anti-tumor and anti-inflammatory^3–7^. In recent years, due to the development of the steroid hormone drug synthesis industry, the demand for diosgenin has been increasing, resulting in the over-exploitation of natural *D. nipponica* resources^8^. *D. nipponica* is mainly propagated by rhizome-division and seedling^9^, but there are many deficiencies of the traditional propagation, such as slow propagation rate, low germination rate, quality degradation and unstable diosgenin yield reduction of the medicinal materials, which are difficult to ensure the urgent demand for rhizomes^10^. In vitro culture could meet for rhizomes demands of commercial uses without any seasonal constraints^11^. Since there are growing concerns over the side effects of chemical medications and the cost of these drugs, more attention has been focused on using natural and plant-derived compounds as an alternative or supplement. Therefore, the willingness to use medicinal plants and plant-derived secondary metabolites is increasing globally. To meet this demand, the cultivation of more medicinal plants or tissue culture to produce more natural products is essential^12^. In vitro regeneration using a combination of plant growth regulators (PGRs) for callus and shoot induction is considered as one of the crucial factors for successful genetic transformation, suspension cell and protoplast culture in plants apart from in planta transformation. Thus, establishing an efficient in vitro regeneration system is essential for the growing demand of *D. nipponica.*

Several reports have demonstrated in vitro regeneration of different species of *Dioscorea. *In vitro plant regeneration is a multi-variable process. Many factors can affect its efficiencies, such as explant type, genotype, concentration and type of PGRs, regeneration medium, and other chemicals that indirectly affect plant growth^13^. The success of plant regeneration in tissue culture is largely dependent on genotype^14^. There are significant differences in the regeneration ability of *Dioscorea* species^15,16^*.* Explant type has a marked effect on shoot regeneration rates in in vitro tissue cultur*e.* Vegetative organs have been used as explants to achieve plant regeneration and tetraploid induction in *Dioscorea zingiberensis* C. H. Wright. Li et al*.*^17^ reported that petiole and stem segments greatly responded to callus induction. PGRs are another important parameter for in vitro plant regeneration. Auxin and cytokinin have been used for induction of regeneration in *Dioscorea deltoidea* and an efficient system from nodal segments of pharmaceutically important plant *D. deltoidea* has been established^18^. However, most papers mainly focused on the screening and optimization of in vitro culture conditions of single kind of explant^19–22^; few studied different explants and mechanism of in vitro morphogenesis in *D. nipponica*. Given the fact that the potential of *D. nipponica* callus in inducing morphogenetic pathways has been rarely addressed, we describe here the morphoanatomical features of different types of callus and determine the location and structure. Moreover, a histological characterization of adventitious bud regeneration is carried out to help understand the mechanisms controlling morphogenic processes^23^. The present study lay the important basis of cytohistology for studying the cell culture of *D. nipponica.*

**Figure 1:**
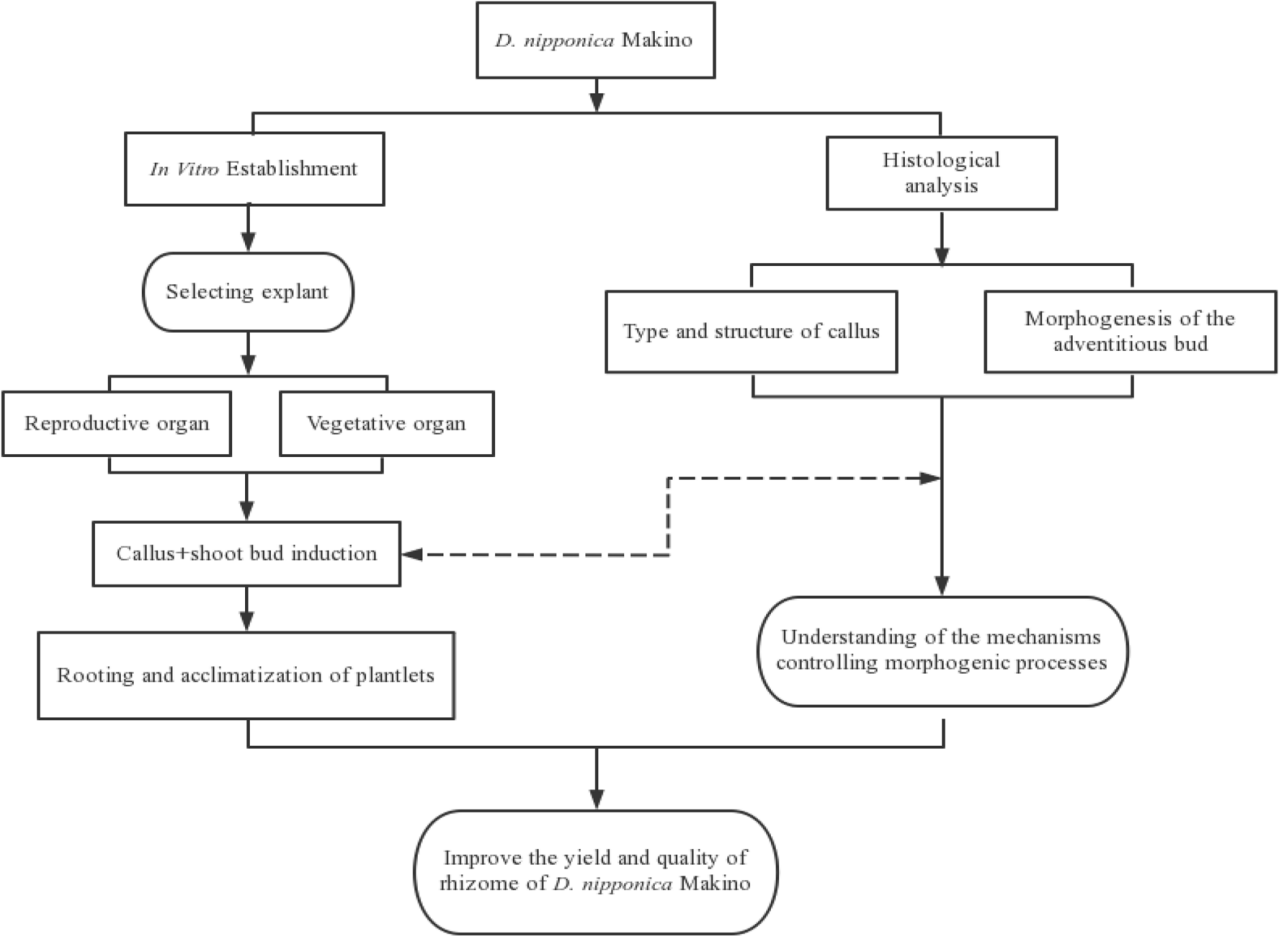
Flow diagram showing the strategy for the experimental manipulations.

In this study, different explants of *D. nipponica* were used with the aim to (1) develop an efficient procedure for callus induction and plant regeneration of *D. nipponica* under in vitro culture conditions. (2) Compare the regeneration effects of different explants. (3) Determine the location and structure of the callus and the origin of adventitious buds. To our knowledge, morphogenic processes of *D. nipponica* have not been reported to date. The strategy for the experimental protocol is summarized in Fig. 1.

## Materials and methods

### Culture medium and growth conditions

MS medium (Murashige and Skoog 1962) was used for all regeneration trails and 1/2 MS medium was used for rooting experiments. Sucrose was used as a carbon source either at 30 g L^−1^. The pH of the medium was adjusted to 5.80 using 1 mol NaOH or HCl and gelled with 7 g L^−1^ agar powder. After pouring the medium into the culture tubes, it was autoclaved at 121 °C for 30 min. After inoculating the explants, cultures were incubated under standard culture room conditions^24^.

### Seed source and explants preparation

**Figure 2:**
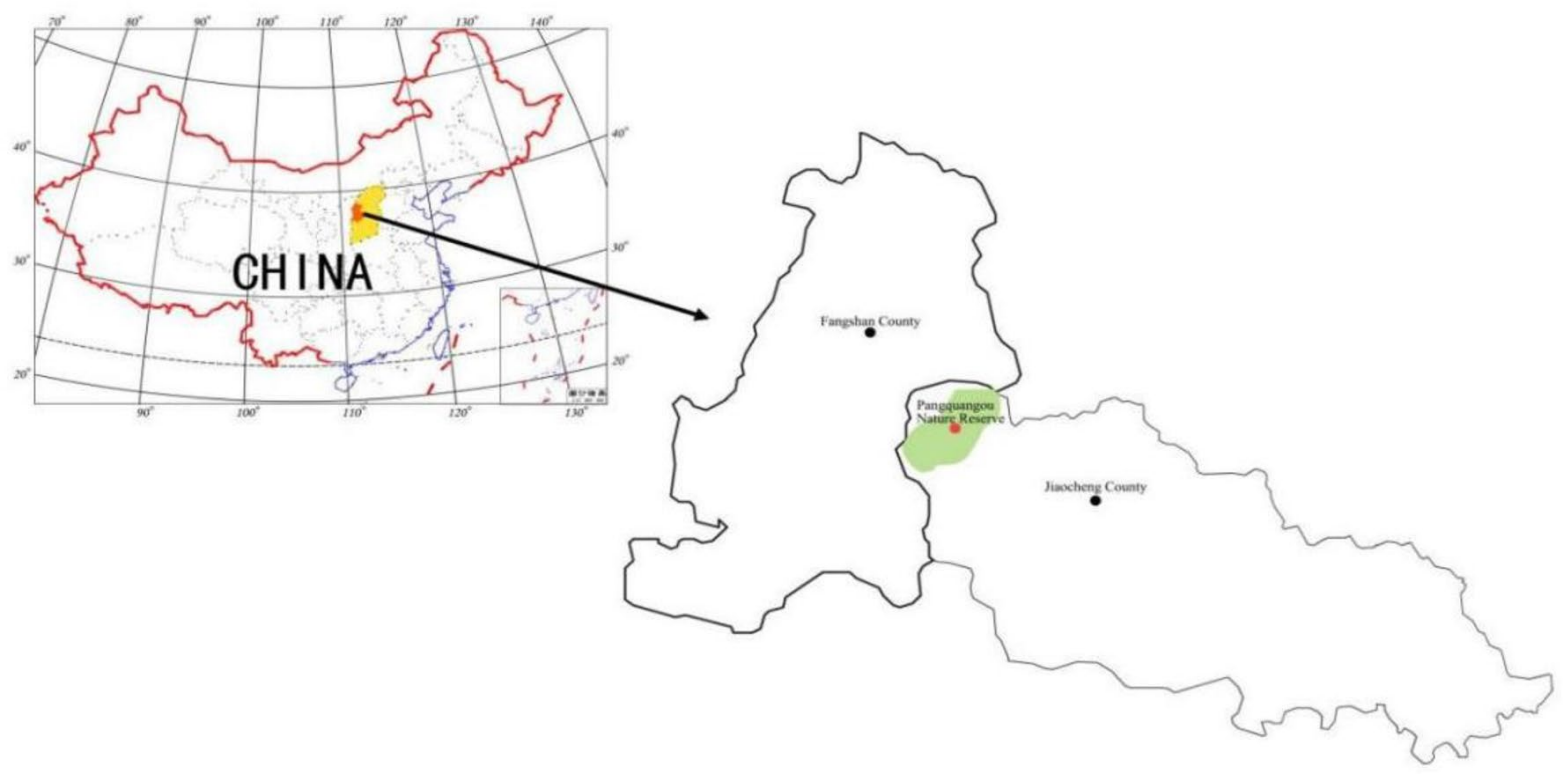
Geographical sites of Pangquangou Nature Reserve, showing geographical range and seed origin.

From September to October 2018, mature seeds of *D. nipponica* were collected in Pangquangou Nature Reserve (37°47′45′′–37°55′50′′ N, 111°22′33′′–111°32′22′′ E) located in the junction of Jiaocheng and Fangshan county, the center of Shanxi Province. The geographical sites are shown in Fig. 2. The specimen was identified by Prof. Runmei Gao College of Forestry Shanxi Agricultural University, and the scientific name was validated online (https://www.plantplus.cn/cn). The voucher specimen was submitted to the Botany Laboratory of Shanxi Agricultural University. Seeds collection were approved by the Shanxi Pangquangou National Nature Reserve Administration. This study meets national and international guidelines for research.

In June 2019, plump seeds were selected, soaked in distilled water for 3 d and transferred to 200 mg L^−1^GA (Gibberellin) solution for 24 h. Seeds were thoroughly washed with saturated detergent solution and running tap water for 30 min^25^. Under aseptic conditions, seeds were surfaced sterilized by immersion in 75% ethanol for 2 min and immediately washing three times with sterilized distilled water, followed by soaking in 2% NaClO for 18 min and rinsing three times with sterilized distilled water. Next, sterile mature seeds were transferred into full-strength MS medium without any PGR for germination. 14 d later, germinating seeds were transferred to autoclaved auxins and cytokinins callus medium.

Vegetative organ: Rhizome coexists with a large number of bacteria and endophytic fungi, which would lead to failure of surface sterilization. In this trial, leaves, stem segments, and rhizomes from aseptic seedlings were used for the regeneration experiments. Leaf explants were cut to an approximately small patch of 0.5 cm^2^. Stem segments and rhizomes were cut into approximately 1 cm in length. Explants were inoculated into a callus medium to induce callus formation.

All the explant materials were placed in the combination of a fresh PGRs medium for clonal propagation at 25 ± 1 °C with a 16/8-h light/dark cycle and light intensity 1500 lx^26,27^. The in vitro trial was treated by a single-factor and two factor block design. Thirty explants were cultured per treatment, with five explants per Petri dish.

### Callus induction and shoot regeneration

For induction of callus from each explant, we used the synthetic auxins NAA (α-Naphthalene acetic acid) or 2,4-D (2,4-Dichlorophenoxyacetic acid), since the effectiveness of those auxins for induction of callus in *D. nipponica* has been previously reported^9,28–30^. Based on those reports, we added 1.0 mg L^−1^6-BA (6-Benzylaminopurine) in combination with varying concentration of NAA or 2,4-D (0.2, 0.5, 1.0 and 2.0 mg L^−1^) to the MS medium for callus induction. Thereafter, high-quality callus was subcultured in medium containing NAA and 6-BA to induce organogenic callus and subsequent shoot regeneration. In addition, basic MS media with different PGRs were compared during the phase of culture, and the optimal media for shoot buds initiation was selected. The percentage of callus induced, shoots developed and the growth state were sequentially recorded after 4 wk of culturing respectively.

Callus induction rate = Number of callus induction /Total number of explants × 100%.

Shoot regeneration frequency = Number of callus regenerating shoots /Total number of callus × 100%.

### Root induction and plantlet transplantation

Half-strength MS medium was supplemented with different combinations and concentrations of NAA (0.1, 0.2, 0.5, and 1.0 mg L^−1^) and 0.5 mg L^−1^6-BA or IBA to optimize root induction medium. Half MS medium without any PGR was used as a control. After 8 wk, plantlets with well-developed roots were removed from the culture bottles and washed to free residues of the medium. A number of roots per seedling was recorded, and the seedling's growth status per PGR combination was also simultaneously observed. Regenerated plantlets were transferred to nutrient soil containing soddy, vermiculite and perlite for 4 wk at 25 ± 2 °C, 75 ± 5% relative humidity, and 12-h photoperiod^31^.

### Histological analysis of callus and adventitious buds

The callus of different textures were collected from the callus induction phase. Here, we describe three types of callus (Callus I-III) with different structure characteristics. Specimens of the callus (Callus I-III) were formed from rhizomes in a medium supplemented with different concentrations tested of PGRs. Callus I-III were observed on medium agitated with 6-BA (0.5 mg L^−1^) and NAA (1.0 mg L^−1^), 6-BA (1.0 mg L^−1^) and NAA (0.2 mg L^−1^), 6-BA (1.0 mg L^−1^) and NAA (1.0 mg L^−1^), respectively.

To look into the developmental stages of bud formation, the specimens of the callus were taken for histological examination. Stems and rhizomes of aseptic seedling were cultured in MS plus 1.0 mg L^−1^6-BA and 1.0 mg L^−1^NAA to induce callus, which was picked every 7 d and cut of 1mm^3^ and fixed in formaldehyde-acetic acid–ethanol fixative (FAA) solution with 70% ethanol for 24 h at 4 °C^32–34^. The trial lasted for 35 d. Sample preparation and the experimental process was carried out using standard procedures described previously by He et al. (2014)^32^.

The embedded sample cut by ultramicrotome and sections (12 μm) were stained with safranin and fast green. Microscopic examination was obtained with a NikonEclipse50i optical microscope, and image-Proplu5.0 was used for measurement and statistics.

### Macro-morphological of rhizomes

Ten plants were randomly chosen to scan rhizomes and acquire digital images by the EPSON Perfection V850 Pro scanner. Macro-morphological indexes of rhizomes, including length, diameter, and volume of rhizomes, were obtained by the WinRHIZO root analysis system.

### Statistical analysis

All data were represented as the mean value for each treatment. Differences among the treatments were determined using analysis of variance (ANOVA). All statistical analyses were performed using SPSS 22.0 software (SPSS, Chicago, IL, USA) and post-hoc testing was carried out using Duncan’s Multiple Range Test. *P* values < 0.05 were considered to be statistically significant.

## Results

### Callus Induction

We examined the effect of the combination of 6-BA and auxin (2,4-D and NAA) on indirect organogenesis from germinating seeds. Callus emerged from the base of the enlarged embryonic axis after 1 wk of inoculation, then after approximately 25 d of culture, callus with shoots gradually formed. Auxin, 2,4-D and NAA are both key phytohormones for the callus induction trail, and the callus induction rate of 2,4-D was significantly higher than that of NAA (Table 1). Moreover, the coloration of the calli on seeds varied: most of the calli were yellowish-green, and their texture was friable and compact when cultured in a medium containing NAA, which also showed a slower growth tendency. After adding 2,4-D, their texture was loose and soft callus appeared and presented a higher growth rate. The optimum hormone ratio for callus induction of seeds was 2.0 mg L^−1^2,4-D and 1.0 mg L^−1^6-BA (Fig. 4A).

**Table 1:** Effects of different concentrations and combinations of 6-BA, NAA and 2,4-D on callus frequency from seeds.

Number	PGRs (mg L^−1^)	Callus number per explant	Relative response (%)
1	6-BA1.0 + NAA0.2	9	30.00^c^
2	6-BA1.0 + NAA0.5	11	36.70^c^
3	6-BA1.0 + NAA1.0	22	73.33^ab^
4	6-BA1.0 + NAA2.0	20	66.70^ab^
5	6-BA1.0 + 2,4-D0.2	16	53.30^bc^
6	6-BA1.0 + 2,4-D0.5	20	66.70^ab^
7	6-BA1.0 + 2,4-D1.0	23	76.70^ab^
8	6-BA1.0 + 2,4-D2.0	25	83.33^a^

Yellowish, loose, and soft callus emerged from the rhizome base after 1 wk of inoculation on MS medium supplemented with auxin, NAA, and 2,4-D (Fig. 4C). In addition, hairy roots also appeared. When the stem segment was used as explants, tender green shoots also emerged from the stem base enlarged and yellowish-green compact callus. But with the culturing duration, some calli were browning and dried out gradually. The leaf was edge-curled, and few white or yellowish-brown particles appeared at the leaf edge after 10 d of inoculation.

As shown in Table 2, callus induction was affected by explant type and hormone ratio. Rhizome showed the highest callus induction rate of 63.33% on medium, adding 1.0 mg L^−1^6-BA in combination with 1.0 mg L^−1^NAA. The induction rate of the stem segment was 43.33% on medium with 1.0 mg L^−1^6-BA and 0.5 mg L^−1^NAA. Leaf was unsuitable for inducing callus, and the induction rate dropped to 16.70%. In addition, the effects of PGRs on callus induction were significantly different between explants of vegetative organs and seeds (Table 2). Callus induction rate of NAA was generally higher than that of 2,4-D with the same concentration (NAA 6.68, 32.51, 45.84%. 2,4-D 2.50, 17.52, 31.86%). But a higher concentration of 2,4-D produced a higher callus induction frequency in seeds callus induction.

**Table 2:** Effects of auxin, NAA, and 2,4-D on different explants callus induction rate.

PGRs (mg L^−1^)			Explant number	Callus induction rate		
6-BA	NAA	2,4-D		Leaf	Stem	Rhizome
1.0	0.2	–	30	0^b^	20.00^ab^	43.33^ab^
1.0	0.5	–	30	10.00^ab^	43.33^a^	46.70^ab^
1.0	1.0	–	30	16.70^a^	30.00^ab^	63.33^a^
1.0	2.0	–	30	0^b^	36.70^ab^	30.00^bc^
**Average**				6.68	32.51	45.84
1.0	–	0.2	30	0^b^	33.33^ab^	53.33^ab^
1.0	–	0.5	30	6.66^ab^	13.33^ab^	26.70^bc^
1.0	–	1.0	30	0^b^	16.70^ab^	36.70^abc^
1.0	–	2.0	30	3.33^b^	6.70^b^	10.00^c^
**Average**				2.50	17.52	31.68

Different letters in the same column indicate significant differences between the treatments (*P* < 0.05).

### Callus induction differences among explants

The callus induction ability and growth state of explants such as seed, leaf, stem segment, and rhizome were compared, and tremendous differences were found (Table 3). No browning was found of seed explants due to less physical damage, resulting in the highest callus induction rate. As to rhizome explants, less callus browning led to a higher callus induction rate. Stem segment explants were also less browning. The browning rate of leaf explants was about 33.33%. Additionally, with the lasting of culturing, the callus observed that the colour changed to brown, followed by necrosis.

**Table 3:** Comparison of callus induced by different explants.

Explant	Callus induction rate (%)	Browning rate (%)	Colour and texture of callus
Seed	83.33	0	Yellowish-green callus with a few hairy roots
Leaf	16.70	33.33	White or yellow–brown callus with granular structures
Stem	43.33	26.70	Compact green callus
Rhizome	63.33	16.70	Yellowish loose callus with large quantity hairy roots

Some explants of rhizomes and stem segments formed not only calli but also shoots, which were derived from the adventitious buds. Callus was also found with obvious morphological differences. Some of calli derived from seeds were found to form shoots and roots simultaneously, and soon whitish fluff appeared from the new adventitious roots. Stem segments were initially basely enlarged, then compact callus appeared and grew green shoots sprouted. Rhizome explants induced yellowish and loose callus, then generated a great quantity of hairy roots and a few adventitious shoot buds. We was cut and removed those shoots derived from original explants when we transferred the explants to induce subsequent organogenesis. Granular callus was induced by leaf explants, taking on slow growth. Among all the treatments, the ability to induce callus of seed and rhizome were stronger than that of stem segment and leaf.

### Bud differentiation

**Figure 3:**
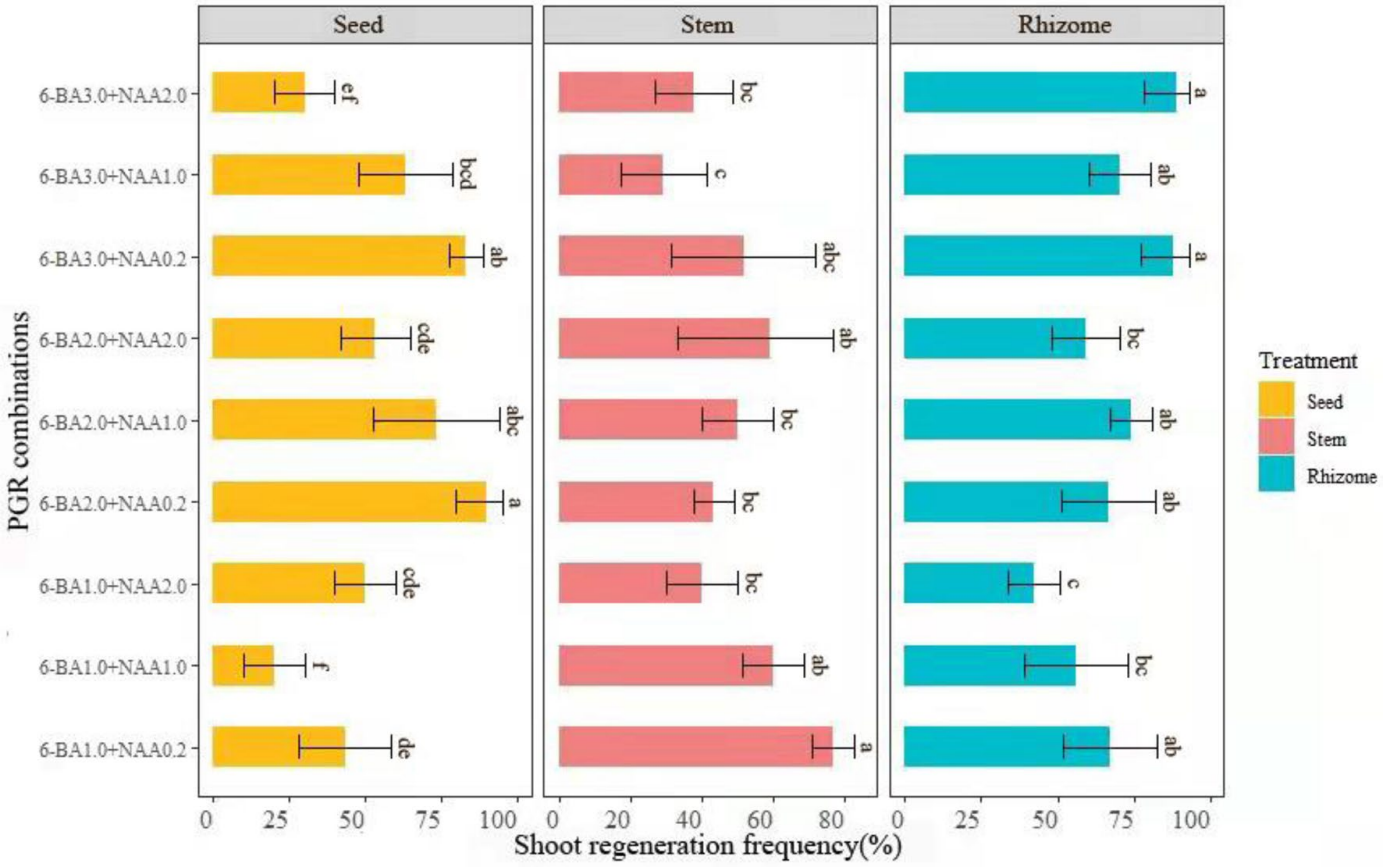
The effect of different concentrations of 6-BA and NAA on the adventitious shoot bud formation from seed, stem, and rhizome callus cultured on MS medium. Values with different letters indicate statistically significant differences (*P* < 0.05) comparing the treatments (differences in combinations of callus bud differentiation hormones of similar origin). Note the log-scaled y-axes.

**Figure 4:**
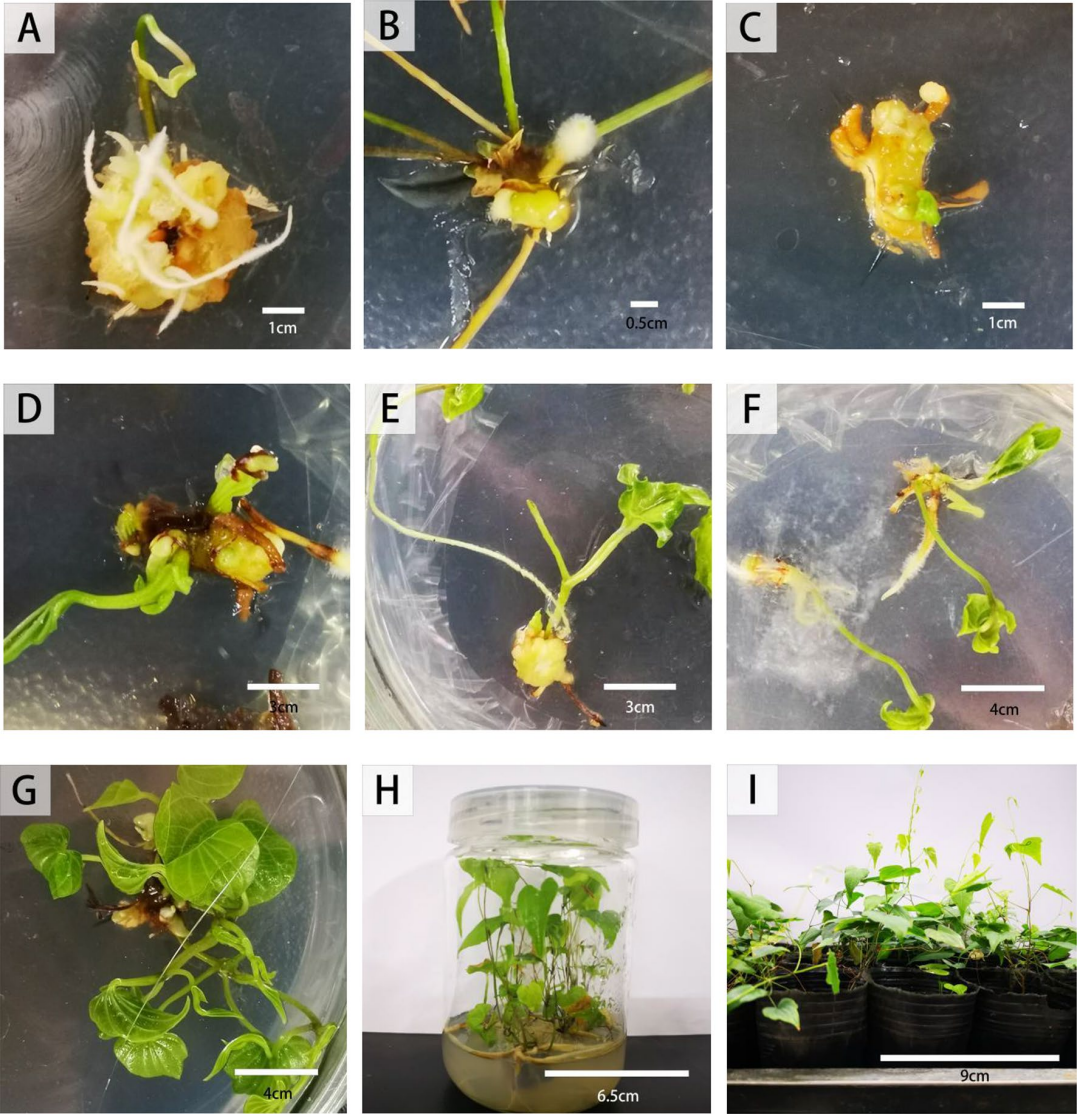
In vitro regeneration from *D. nipponica*. (**A**–**C**) presented callus formed by seed, stem segment, and rhizome explants, respectively; D and E presented shoot initiation of rhizome-induced callus, the (**D**) presented the combination of 3.0 mg L^−1^6-BA + 2.0 mg L^−1^NAA induced shoot, the (**E**) presented the combination of 3.0 mg L^−1^6-BA + 1.0 mg L^−1^NAA induced shoot; (**F**) presented hairy roots of adventitious buds; (**G**) presented elongation and multiplication of regenerated shoots; (**H**) presented rooting of shoots; (**I**) presented regenerated plant transferred to nutrient soil.

It is necessary for exogenous application to induce adventitious shoot buds. Further, shoot regeneration from the callus was achieved by supplementing 6-BA (1–3 mg L^−1^) and NAA (0.2, 1.0, and 2.0 mg L^−1^) into MS medium (Fig. 4D–G). Microshoots and hairy roots emerged from the base of the callus within 3 wk (Fig. 4F). Effects of 6-BA and NAA on the formation of adventitious shoot buds were shown in Fig. 3.

Callus from seed, stem segment and rhizome showed various differentiation abilities. Most calli were found of bud protuberance on the surface within 5 d, and further developed into shoots 10 d later. A few calli also simultaneously produced hairy roots (Fig. 4A). The frequency of adventitious shoot buds regenerating explant varied between 20 and 90%, being higher from callus of seeds and rhizomes. The best results were obtained when callus derived from seeds were cultured on MS medium addling 2.0 mg L^−1^6-BA and 0.2 mg L^−1^NAA. In such conditions, the regenerating explants also grew vigorously (Fig. 4G). Followed by the rhizome-induced callus, maximum shoot differentiation frequency (88.57%) was achieved by supplemented with 3.0 mg L^−1^6-BA and 2.0 mg L^−1^NAA (Fig. 4D). The formation of shoots from callus derived from stems treated by hormone combinations of group 3.0 mg L^−1^6-BA and 1.0 mg L^−1^NAA, 3.0 mg L^−1^6-BA and 2.0 mg L^−1^NAA (Fig. 3) was significantly less than other combinations.

### Root formation

Regenerated shoots (length of 30–40 mm) were separated from multiple shoot clusters and transferred to a half-strength MS medium augmented with various concentrations of different PGRs. White root primordia appeared after 10 d culturing, then developed into young adventitious roots about 20 d later. The effect of hormones on rooting is presented in Table 4. A maximum rooting frequency (86.70%) and an approximate 13.20 roots were achieved after 6 wk of culturing on half-strength MS medium supplemented with 0.5 mg L^−1^NAA (Fig. 4H). In such conditions, the regenerating plants also showed better growth with robust roots, dark green and larger leaves. As 0.5 mg L^−1^6-BA additionally added, the number of regenerated roots was 10.70 and the rooting frequency was 73.33%, which was close to single addition of 0.1 mg L^-1^ NAA. However, the regenerating plant of the former group grew well; the latter group had relatively delicate stems and small yellow-green leaves. When 0.5 mg L^−1^6-BA and 1.0 mg L^−1^NAA were added into the medium, the numbers of roots decreased significantly, but its rooting frequency was still significantly higher than control.

**Table 4:** Effect of PGRs and their concentrations on roots formation frequency and number of roots.

PGRs (mg L^−1^)			No. of roots per shoot	Relative response (%)	Growth status of rooting seedlings
NAA	6-BA	IBA			
–	–	–	7.4 ± 1.90^c^	33.33 ± 4.70^c^	Small leaves in yellow-green, thin stems, more fibrous roots
0.1	–	–	10.5 ± 1.27^b^	66.70 ± 17.00^ab^	Small leaves in yellow-green, thin stems, obvious taproots, more lateral roots
0.5	–	–	13.2 ± 2.15^a^	86.70 ± 4.71^a^	Thick leaves in dark-green, stout stems, obvious taproots, more lateral roots
0.5	0.5	–	10.7 ± 1.64^b^	73.33 ± 12.47^ab^	Small leaves in dark-green, slender stems, obvious taproots, more fibrous roots with whitish fluff
1.0	0.5	–	8.7 ± 1.06^c^	60.00 ± 8.16^b^	Small leaves in yellow-green, delicate stems, no obvious taproot, more fibrous roots
0.2	–	0.5	3.4 ± 0.84^d^	26.70 ± 4.71^c^	Large leaves in yellow-green, stout stems, no obvious taproots, more lateral roots

The data represented mean ± standard error; Different letters within different root induction mediums indicate significant differences (*P* < 0.05) comparing the treatments.

### Plant transplantation

The acclimatization of in vitro regenerated plantlets was a difficult step of the micro-propagation protocol establishment of their susceptibility to fungal diseases^35^. In the present study, well-rooted micro propagated plantlets were removed from the medium, washed, and successfully transplanted into small plastic pots containing sterile soil rites. After 4 wk of growing in a greenhouse, young plants grew vigorously and eventually established in soil with 80% survivability (Fig. 4I).

### Callus type and its structure characteristics

**Figure 5:**
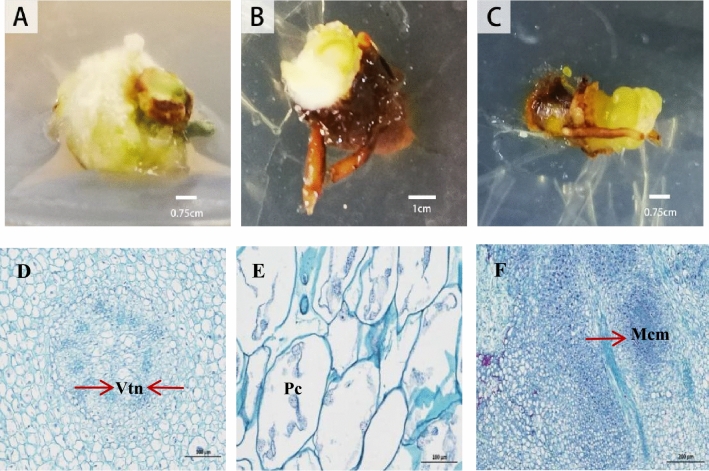
Three types of callus and their cytological observation. (**A**,**D**) external morphology and internal cell structure of callus type I; (**B**,**E**) external morphology and internal cell structure of callus type II; (**C**,**F**) external morphology and internal cell structure of callus type III. (Vtn-Vascular tissue nodules; Pc-Parenchyma cell; Mcm-Meristem cell mass).

There were morphological differences among callus induced by rhizome. According to obvious color, morphology and texture differences, the callus could be divided into three types (Fig. 5A–C). Callus type I: whitish in color, compact and puffy texture. Callus type II: yellowish or whitish in color and loose structure. Callus type III: greenish in color and a nodule-like protrusion surface.

Microscopical observation revealed the cell structure of the above three types of callus (Fig. 5D–F). The callus was externally covered with meristem, and a large number of parenchyma cells with small nuclei can be seen in the internal structure (Fig. 5E). There was also a few of vascular tissue nodules can be seen, which was of the uneven thickening cell wall, nest-like structure (Fig. 5D). The vascular system could be seen from the cross-section of the three types of callus. In callus type I, more vascular tissue nodules of tightly arranged cells were found, with a great quantity of tracheids, and clustering gathered meristematic cells inside callus. As to callus type II, more big parenchyma cells with small nuclei were regularly distributed and in slices, but fewer vascular tissue nodules were seen in the callus. Abundant vascular tissue nodules were also seen in type III of the callus, which was full of the most closely arranged cells with less interstitial substance and well-developed tracheids, meristematic cells gathered in clusters on the surface and interior of the callus. Contrasted to the callus type I and II, the cell differentiation ability of callus type III was the strongest, and the cells were most closely arranged (Fig. 5F).

### Morphogenesis of the adventitious bud

**Figure 6:**
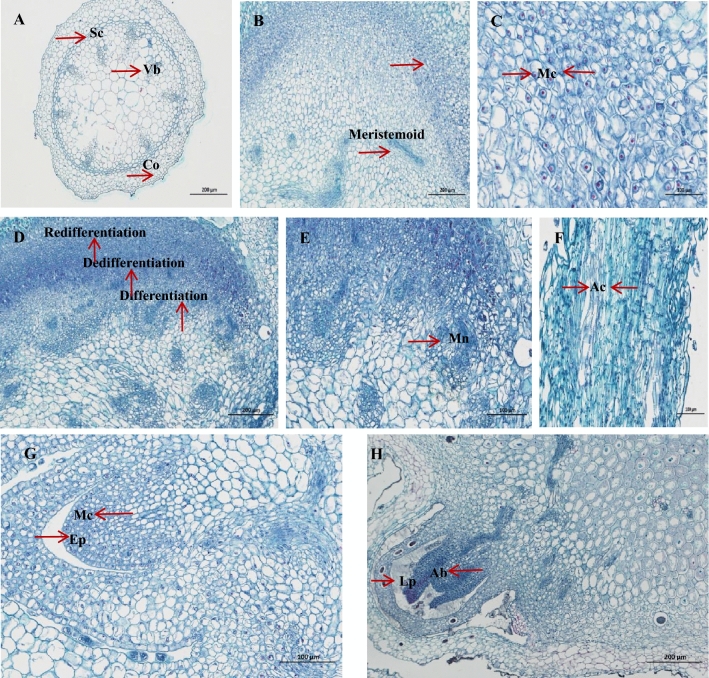
Histological analysis of the adventitious bud of *D. nipponica.* (**A**) Caulicle structure of aseptic seedling. (**B**) Dedifferentiation of cortical parenchyma cells. (**C**) Meristematic cells in callus. (**D**) Meristematic nodule in callus. (**E**) Globular meristematic masses. (**F**) Vascular tissue in callus. (**G**) Formation of bud primordia at the near-surface of callus. (**H**) Fully developed shoot bud with bud primordium and leaf primordium, arrow. (Mn-Meristematic nodule; Ac-Annular catheter; Mc-Meristematic cell; Ep-Epidermis; Lp-Leaf primordium; Ab-Adventitious bud.).

Histological examination confirmed indirect shoot regeneration of *D. nipponica* (Fig. 6B–H). Features of the stem were observed in cross sections of initial explants (Fig. 6A), including epidermis, collenchyma, sclerenchyma, and vascular bundles scattered throughout the parenchyma cells. The optical microscope study of *D. nipponica* found that the callus formation underwent three stages: initial, mitotic, and formation. Under the action of exogenous hormones, the stem segments were dedifferentiated, divided, and proliferated into cell masses and meristemoids (Fig. 6B), then formed a large number of callus, which distributed on the surface of the explants, thus completing the dedifferentiation process.

The differentiation of the callus was observed. There were many meristematic cells in the callus induced by stem segments. The meristematic cells divided in all directions and gradually formed structure, representing the onset of the establishment of meristemoids that were massed by small cells with large nuclei (Fig. 6C,D). About 10 d later, morphologically larger globular meristematic masses were produced, called meristematic nodules (Fig. 6E), which divided and differentiated again, and the primitive of organ primordia appeared. With the elongation of buds, parenchyma cells beneath the growth tip differentiated into tracheary elements (TEs), which formed vascular tissues and gathered into nodules. Vascular tissue nodules were network connected with other surrounding tissues (Fig. 6F) to fulfill the transportation of water and nutrients. The callus at the bottom was well connected with the apical meristem at the top, characterized by adventitious buds (Fig. 6G–H). It showed that the bud differentiation of *D. nipponica* callus was of exogenous origin and mostly occurred the near-surface of the callus.

### Morphological changes of rhizome

**Figure 7:**
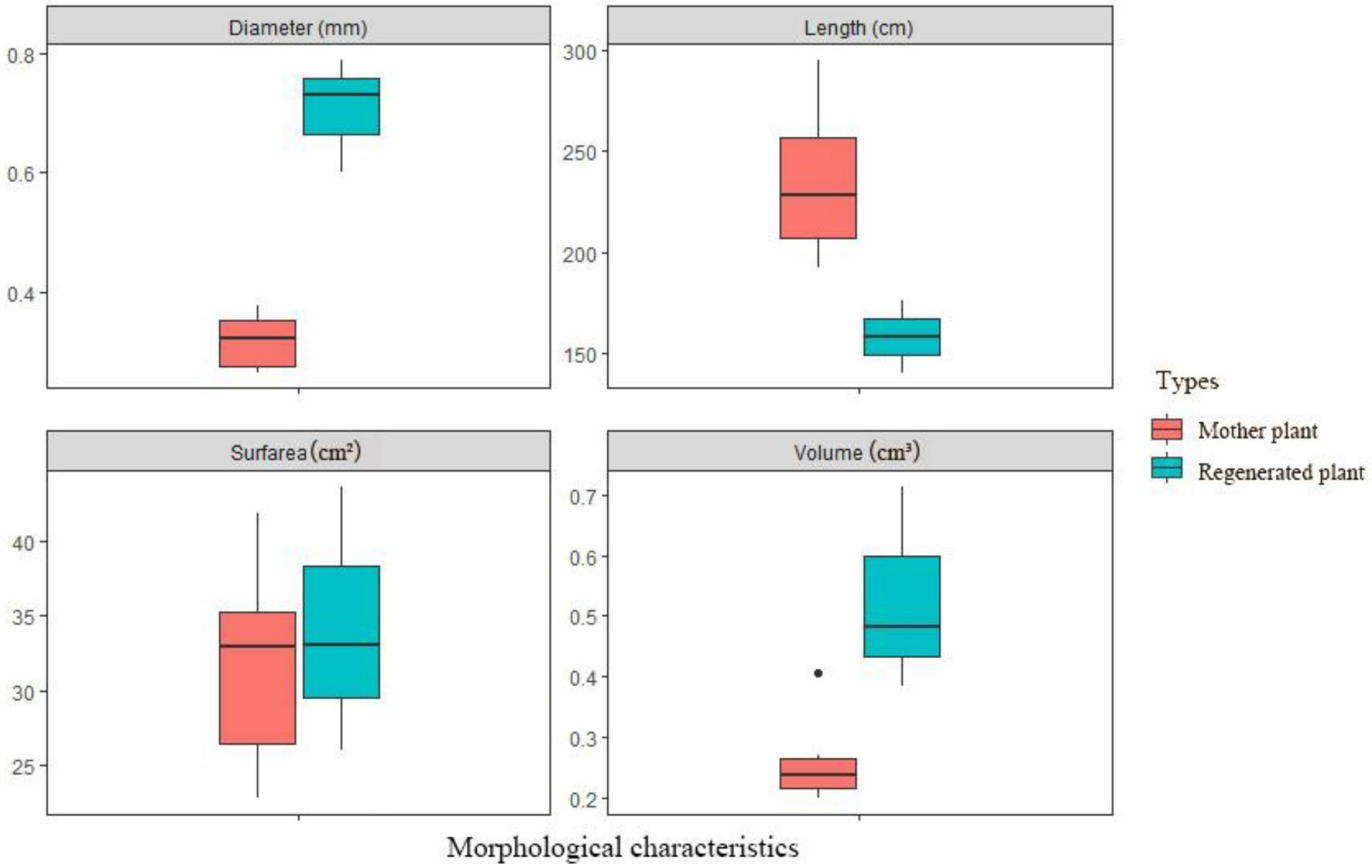
Box-whisker plots for two types of Morphological characteristics in rhizomes both inventories with median and inter quartile ranges; whiskers extend to 1.5 times the inter quartile range. Note the log-scaled y-axes.

Morphological characteristics of mature plants, both micropropagated and seed-propagated means, were recorded. Leaves of seed propagated plants and in vitro plants showed similar morphological characteristics (data not shown), but rhizomes illustrated some significant differences (*P* < 0.05). Both the diameter and volume of rhizomes were significantly higher in the regenerated plants, but the length showed an opposite tendency (Table 5, Fig. 7). Length in seed propagated plants were mostly in the range (192.03–295.08 cm), whereas the in vitro cultured plants were only in the range (140.22–176.08 cm). The rhizomes of in vitro cultured plants were more stout, with slightly enhanced diosgenin content (data not shown). While rhizomes of seed propagated plants were slender and lateral roots developed. It indicated that exogenous addition of PGRs might influence the accumulation of secondary metabolites.

**Table 5:** Morphological traits in rhizomes of seed propagated and in vitro plants under greenhouse conditions.

Plant types	Length (cm)	Surf area (cm^2^)	Diameter (mm)	Volume (cm^3^)
In vitro regenerated plant	158.11 ± 14.64**	34.24 ± 7.26	0.71 ± 0.08*	0.53 ± 0.14*
Seed propagated plant	235.71 ± 39.00**	31.81 ± 6.51	0.32 ± 0.04*	0.26 ± 0.07*

The data represented mean ± standard error; indicated significant differences between the morphological indexes for the two types are shown** at the 0.01 level;* at the 0.05 level.

## Discussion

### Explants selection on callus formation

Callus induction is important to establish a plant regeneration system and genetic transformation. It could be widely applied in rapid reproduction, breeding, transgenic and virus-free seedlings production^36,37^. The organogenesis ability of different tissues of plants varies greatly. The selection of explants is a key factor in the tissue culture system. The order of induction rate of different explants for *Dioscorea opposita* Thunb was once reported that bulbil had the highest amount of callus while leaf and tuber produced the lowest amount^38^. Peng et al*.* (2009)^29^ found that all kinds of vegetative organs of *D. nipponica* could induce callus, but the explants had different degrees of browning, which seriously affected callus growth. This study investigated the most suitable explant source and combination of PGRs for callus induction and the best concentration of 6-BA for shoot production. Seeds could induce a large number of fast-growing calli because of no physical damage. However, before the inoculation of the callus induction medium, the seeds had to be tiled in the MS medium first. Otherwise, the callus induction rate was very low. Callus induction with 2.4-D would interfere with the distribution pattern of auxin and strengthen the auxin effects on the whole embryo, which might inhibit the formation of plant embryonic axis^39^. Therefore, seeds were placed into the MS medium to develop bud and embryonic axis and then inoculated into the callus induction medium. Until now, most in vitro cultures of *D. nipponica* only used a single explant. We selected vegetative organs of aseptic seedlings as explants, considering of lessening pollution of endophytic fungus in rhizomes. The callus induction ability of seed and rhizome was higher than that of stem segment and leaf, which might be due to different contents of endogenous (cytokinin and auxin) hormones during in vitro culture^40^. The response of explants to hormones is closely related to the physiological state of the material itself, the diversity of plant receptors, endogenous hormone synthesis, and metabolism differences^41,42^.

### PGRs on in vitro culture

PGRs play an important role in the induction and differentiation of callus for species of *Dioscorea*. Because the induction rate and plant growth vary greatly among different explants, the type and concentration of PGRs must be changed accordingly. In 2007, in vitro of *D. nipponica* was successfully accomplished by induction of shoots and microtubers, indicating that the synergism of BA and NAA was extremely favorable for tissue culture^8^. Among PGRs, auxins and cytokinins are the main determinants for in vitro test-tube seedlings to form shoots and produce roots. The type and range of organogenesis in cell culture depend on the ratio of auxin to cytokinin^43,44^. Cytokinins such as Kn (Kinetin) and 6-BA have been shown to promote cell division, bud proliferation, bud morphogenesis and inhibit root formation, whereas auxins such as NAA and 2,4-D are commonly used to stimulate callus production and cell growth, induce somatic embryogenesis as well^42,45–47^. Callus induction in several Dioscoreaceae members has been achieved using different concentrations and combinations of auxins. In *Dioscorea Tokoro* Makino, 1/2MS medium supplemented with 10 µM 2,4-D for callus induction^48^, whereas in *D.zingiberensis*, a combination of 1.5 mg L^−1^ 2,4-D and 1.0 mg L^−1^ 6-BA induced prolific callus from stem explant^49^. A combined effect of 1.0 mg L^−1^ 6-BA and 4.0 mg L^−1^ 2,4-D in MS medium induced maximum callus formation from stem tip in *D. nipponica*^28^indicating varied response for callusing in different plant species to plant growth regulators. Auxin is the most effective PGR for callus induction and cell growth for plant species^50–53^, while the effects would be reduced when used singly. Therefore, cytokinins (6-BA) and auxins (NAA and 2,4-D) were selected in our experiment at all stages of tissue culture for *D. nipponica*. Especially in roots cultured, half MS medium containing 0.5 mg L^−1^NAA and 0.5 mg L^−1^6-BA plus 0.5 mg L^−1^NAA resulted in earliest as well as plenty of lateral root formation. Several reports are available in the literature indicating the effect of lower concentration of NAA exhibiting synergistic effect along with BA and increased shoot regeneration from callus of *Dioscorea*; shoot bud regeneration from stem tip callus *D. nipponica*^28^; and also in direct regeneration from *D. composita*^42^. Our results of the synergistic effect of cytokinin combination and low auxin concur with reports of *D. zingiberensis*, a member of the same family^54^.

### Histological examination of regeneration pathway

In in vitro culture of plants, although there are differences between plant materials and regeneration pathways (indirect organogenesis or somatic embryogenesis), all of them are initiated by cells in specific parts of the explants, which divide to form a callus, produce embryogenic cells, and then go through the process of cell dedifferentiation^55^. Plant cells and tissues cultured in vitro can be redifferentiated by dedifferentiation. In other words, adventitious buds were produced from the callus by cell differentiation^56^. Little attention has been paid to the histological characterization of the regeneration pathway of *D. nipponica* explants. Our research found that under in vitro culture conditions, vascular tissue first initiated division, then parenchyma cells divided and proliferated into cell masses to come into being meristematic zone. Further, the cells in the meristematic zone underwent division in all directions to form the structure. Afterward, morphologically larger meristematic nodules were produced, which became the growth center or vascular tissue nodules for nutrient transportation. Bud differentiation was mostly of exogenous origin, and mainly near the callus surface, which was in agreement with previous findings of other plants^57,58^. Therefore, it may be surmised that in vitro morphogenesis of *D. nipponica* was mainly caused by indirect organogenesis (adventitious bud). There were obvious differences between the three types of callus induced by rhizomes. In particular, callus type III were nodule-like, puffy, and compact, preferred for subsequent shoot induction because it produced the strongest differentiation ability of cells. There were also more bud primordia than in callus I and II, indicating that the vascular tissue nodules and meristem cells in callus III were closely related to bud differentiation. The reason for this is that the morphogenesis ability of the callus is perhaps related to the physiological maturity of the explants^59^. Due to the interaction between physiological maturity and exogenous hormone sensitivity, the levels of meristematic cells produced during callus formation are different, and the meristems themselves are also different, which in turn affects callus formation. The causes of the differences in the cell structure of the three types of callus need to be further investigated.

## Conclusions

In vitro plant regeneration of *D. nipponica *via indirect regeneration using different explants is reported. This protocol describes a successful rapid regeneration system on MS medium using a combination of 1.0 mg L^−1^6-BA and 2.0 mg L^−1^2,4-D for callus-mediated indirect regeneration from seed explants. In such conditions, the callus induction rate amounted to 83.33%. When rhizome explants were cultured for 4 wk on MS medium plus 1.0 mg L^−1^6-BA and 1.0 mg L^−1^NAA, a higher callus rate of 63.33% was achieved. Calli derived from seeds, stems, and rhizomes of *D. nipponica* effectively differentiate shoots. Maximum shoot differentiation frequency of 90% was observed in callus derived from seeds on MS medium with 2.0 mg L^−1^6-BA + 0.2 mg L^−1^NAA, being the optimum combination. The shoot differentiation frequency (88.57%) of rhizome-induced callus was obtained by the combination of MS medium supplemented with 3.0 mg L^−1^6-BA + 2.0 mg L^−1^NAA. 1/2 MS medium plus 0.5 mg L^−1^NAA resulted in a higher root regeneration frequency of 86.70%. Histological analysis showed that type III calli comprised a dense-arranged and rapidly-growing cluster of cells, implying stronger cell differentiation ability. Adventitious buds were initiated near the surface of the callus, which is the characteristic of the indirect organogenic process.

Hairy roots are characterized by rapid growth, high content of secondary metabolites, high genetic stability, and industrial production potential, which can realize industrial production of secondary metabolites in medicinal plants^13,60^. In our experiment, hairy roots were produced by explants except for leaves during the callus proliferation and bud differentiation process. It was reported that *Agrobacterium* *rhizogenes* can induce hairy roots, studies focused on diosgenin content and stimulation of hairy roots would be carried out. Moreover, the indirect regeneration pathway via callus culture opens a new way of genetic transformation to accomplish the industrialization of secondary metabolites for *D. nipponica*.

## Data Availability

The datasets generated during and/or analyzed during the current study are available from the corresponding author on reasonable request.

## Abbreviations

PGRs: Plant growth regulators
6-BA: 6-Benzylaminopurine
NAA: α-Naphthalene acetic acid
2,4-D: 2,4-Dichlorophenoxyacetic acid
IBA: Indole-3-butyric acid
Kn: Kinetin
GA: Gibberellin
